# Assessing mental health in a context of extreme poverty: Validation of the rosenberg self-esteem scale in rural Haiti

**DOI:** 10.1371/journal.pone.0243457

**Published:** 2020-12-14

**Authors:** Keetie Roelen, Emily Taylor

**Affiliations:** 1 Institute of Development Studies, Brighton, United Kingdom; 2 The University of Edinburgh, Edinburgh, United Kingdom; Universidad Loyola Andalucia Cordoba, SPAIN

## Abstract

A widening evidence base across low- and middle-income countries (LMICs) points towards mutually reinforcing linkages between poverty and mental health problems. The use of validated and culturally relevant measures of mental health outcomes is crucial to the expansion of evidence. At present, there is a paucity of measures that have been tested and validated in contexts of extreme poverty. Using data from adult women living in extreme poverty in rural Haiti this study assesses the cross-cultural validity of the widely used Rosenberg Self-Esteem Scale (RSES) and its applicability in assessing linkages between poverty and mental health outcomes. We find no evidence for a one-dimensional 10-factor structure of the RSES within our data and agree with other authors that the standard self-esteem model does not fit well in this cultural context. Comparisons with another widely used measure of mental health–the K6 measure–indicate that the RSES cannot be used as a proxy for mental health outcomes. We conclude that the use of the RSES in different cultural contexts and with samples with different socioeconomic characteristics should be undertaken with caution; and that greater consideration of the validity of psychosocial constructs and their measurement is vital for gaining robust and replicable insights into breaking the cycle between poverty and mental health problems.

## Introduction

Despite widespread poverty and high levels of mental health disorders, research on the relationship between poverty and mental health in low and middle-income countries (LMICs) has only started emerging in the last two decades [1,2]. Evidence from across LMICs finds an association between indicators of poverty such as low socioeconomic status and food insecurity, and common mental health disorders [2]. While some studies suggest that poor mental health is not strongly associated with poverty [3,4], others have dispelled this by attributing lack of association to narrow or inadequate use of poverty measures [5,6]. More recent research in countries including India and Indonesia points towards a causal relationship between low income and poor mental health [7,8]. Nevertheless, research on linkages between poverty and mental health, and particularly the role of poverty alleviation interventions in improving mental health, in low-resource settings is scarce [1].

The use of validated, comparable and culturally relevant measures of mental health outcomes is central to the expansion of research. Efficiency and generalisability are key requirements of such measures, particularly when mental health is not the primary focus of enquiry but one of a broad set of socio-economic outcomes, as is common in relation to anti-poverty interventions [9,10]. Despite a widening of the evidence base, there is a paucity of measures that have been validated within the context of LMICs and that ensures their relevance in different resource and cultural contexts [11,12]. Against the backdrop of this paucity, we assess the validity of a widely used measure of self-esteem in a context of low resources and high levels of extreme poverty in rural Haiti.

Self-esteem describes an individual’s positive or negative evaluation of their self-worth, self-confidence and self-respect [13]. Self-esteem is positively associated with goal-directed behaviour [14], negatively associated with depression [15] and a wide range of other psychiatric disorders [16]. Evidence leans towards self-esteem being an etiological factor in the development of depression (the vulnerability model) rather than a side-effect (the scar model) of depression [17]. Therefore, self-esteem is not only an indicator of psychological wellbeing [18] but may also serve as an early indicator of vulnerability to depression or other psychological distress. The Rosenberg Self Esteem Scale (RSES) [19] is regarded as the gold-standard measure of self-esteem, with established reliability and validity, and is used across different contexts, languages and cultures [20].

In this paper, we use data from adult women living in extreme poverty in rural Haiti to assess the cross-cultural validity of the RSES, and its applicability in studies on linkages between poverty and on mental health outcomes. For this purpose, we investigate two specific questions in reference to our data and context: (i) Does a Creole-language version of the RSES have a coherent factor structure when applied in Haiti? and (ii) Can the RSES serve as a proxy for mental health? We do so by investigating the factor structure of the RSES and by comparing results for the RSES with another measure of mental health in order to establish construct validity, namely the K6 [21].

## RSES in LMICs

We provide a short review of applications of the RSES in LMICs.

The RSES was originally designed as a single-dimension construct [22] measuring self-esteem via 10 items and a four-point scale. Items cover what are understood to be universal indicators of self-esteem such as self-worth, self-respect and self-like using both positively and negatively worded items (more detail is provided in the section Measures below). A comparative study of the RSES in 53 nations, including 10 LMICs, found broad statistical support with a one-factor model [20]. Other country-focused studies, such as in Brazil, also find high internal consistency [23]. However, the amount of variance accounted for by the single factor was low in some countries, especially in Botswana, the Democratic Republic of Congo (DRC) and Ethiopia, where it fell below 30 percent. This was reflected in poor internal consistency as measured by Cronbach’s alpha in the DRC, Ethiopia, and Tanzania [20].

Confirmatory factor analyses (CFA) provide a more thorough validation of a scale’s internal structure. We found five studies using CFA with samples from LMICs, describing six samples (see Table 1). Only Fromont et al [24] found support for a one-factor model, in Burundi, with weak internal consistency (α = .63). All other studies found that a two-factor model achieved the best fit across multiple indices. Two studies excluded item 8 to achieve the best fit, resulting in a 9-item scale [22,25]. Both these samples were Chinese, but another study with a Chinese sample preferred a 10-item model [26].

**Table 1:** Published confirmatory factor analyses in LMIC-derived samples.

1^st^ author	LMIC & Language	Sample details	N items	N Factors	CFI^1^	RMSEA^2^	Cronbach’s α^3^
Fromont (2017)	Burundi—French version translated into Kirundi	Burundi health workers and general population N = 906	10	1	0.966	0.045	.63
Makhubela (2017)	South Africa–NR^4^	Black South African students n = 579	10	2	0.988	0.032	.73
Wu (2017)	China—Chinese	Migrant and urban children in China N = 982	10	2	0.995	.036	.73
Li (2015)	China—Chinese,	Adolescents n = 350	9	2	0.988	.072	.84
	Costa Rica–Spanish	Adolescents n = 343	10	2	0.989	.046	.76
Farruggia (2004)	China–Chinese	Adolescents N = 502	9	2	0.966	.065	.83

^1^Comparative Fit Index.

^2^Root Mean Squared Error of Approximation.

^3^Internal consistency of scale prior to CFA.

^4^Not reported.

Two features recurred across these studies. Firstly, method effects were common across samples with items splitting into two factors depending on whether they were negatively or positively worded. Secondly, item 8 “*I wish I could have more respect for myself*” seems to have been variously interpreted by different cultures, with the consequence of it not reliably loading onto the one-factor model or onto the negatively worded factor in the two-factor version. This does not appear to be a language translation issue, as the phenomenon has been noted in English-speaking LMICs such as Botswana and Zimbabwe [20]. These features suggest a cultural effect. The differential understanding of item 8 has contributed to an argument that the effect is in some way caused by collectivist culture, although this argument has been applied to countries that would not fit the typically understood collectivist paradigm. Wu, Zuo [26] argued that item 8 should be treated as a positively worded item. However, in other studies item 8 does not appear to be a useful part of the scale construct, and may be measuring something different altogether.

Arguments about the cultural specificity of self-esteem more generally tend to be polarised. Hewitt [27] argued that self-esteem is essentially socially constructed and therefore culturally situated. Du, King [28] point out that individualistic cultures may place more emphasis on personal self-esteem while relational aspects of self-esteem may be more salient in collectivist cultures. At the other end is the position of self-esteem as a trait characteristic of humans and therefore universal [29]. The possible cultural specificity of self-esteem, and therefore the RSES, aligns with a wider literature cautioning against comparing mental health outcomes across interventions and contexts without ensuring that construct and measurement of it are applicable to that culture [30]. The picture is further complicated by the assertion that self-esteem is gendered, with large-scale cross-cultural evidence showing that men consistently have higher self-esteem than women [31]. Researchers should therefore approach self-esteem measurement in specific populations with caution, with routine validation within samples preliminary to other analyses. However, evaluations of anti-poverty interventions in different LMICs, having employed the RSES as a measure of psychological wellbeing, tend not to report steps to establish sample-level validation such as confirmatory factor analysis [10,32,33].

This study therefore set out to validate the RSES in a sample of Haitian women, testing construct validity using confirmatory factor analysis and discriminant validity through comparison with a measure of mental health, namely K6. Secondly, we aim to test whether self-esteem, as measured by the RSES, can be used as a proxy measure for mental health, hypothesising that self-esteem will predict mental health.

## Methods

### Design, sample and procedure

The data is drawn from the baseline study of a quasi-experimental impact evaluation of the *Chemen Lavi Miyò* (CLM)—“the pathway to a better life”–programme in rural Haiti, which is implemented by local NGO *Fonkoze*. The CLM programme targets adult women who are extremely poor [34] and supports them with a package of cash and asset transfers, skills development, coaching and service provision over a period of 18 months in a bid to move them out of poverty [35].

The data includes 1,381 women from across treatment and control groups in the Central Plateau region in Haiti. The sample for the treatment group (n = 631) was pre-determined by programming considerations, with all women in the programme sites who were eligible having been selected into the programme. Inclusion criteria include living in extreme poverty (based on a wide set of indicators such as having little income, being unable to send their child(ren) to school and having limited assets), having dependants and being able to work. Women from similar communities in the same region were selected into the control group (n = 750) using participatory wealth rankings (PWRs) within selected communities. PWRs are widely used participatory and community-based exercises that ask a small group of community members to rank those living in the community according to their wealth, serving as a proxy for poverty status and helping to establish programme eligibility.

The two sub-samples are described in Table 2 for illustrative purposes. Women had an average age of 33.49 years with median household size of 5 members, including median of 3 children under 18 years and 1 child under 5 years. More than three out of four women were traditionally or legally married. Participants’ literacy was ranked on a 4-point scale from completely illiterate to able to read and write; 67 percent were unable to read or write. Although differences between groups in household size, numbers of children and marital status reached significance, effect sizes were negligible (Cohen’s d<0.2, r<0.2).

**Table 2:** Overview of sample.

Characteristic	measure of central tendency/%	Total sample (n = 1381)	Treatment group (n = 631)	Control group (n = 750)	p-value	effect size
Age	Mean (SD)	33.5 (11.7)	33.6 (11.8)	33.4 (11.6)	.647	0.03
N children	Median (IQR)					
aged 0–5		1 (0–2)	1 (0–2)	1 (0–1)	< .001	0.10
aged 0–18		3 (2–4)	3 (2–4)	2 (1–4)	< .001	0.11
Household size	Median (IQR)	5 (4–6)	5 (4–6)	5 (3–6)	< .001	0.10
Marital status	N (%)				< .001	0.17
Never married		104 (7.5)	19 (3.0)	85 (11.3)		
Traditionally or legally married		1069 (77.4)	515 (81.6)	554 (73.8)		
Divorced/separated		129 (9.3)	51 (8.1)	78 (10.4)		
Widowed		69 (5.3)	39 (6.2)	30 (4.0)		
Literacy	Median (IQR)	0 (0–1)	0 (0–1)	0 (0–1)	.006	0.12
RSES total	Mean (SD)	15.6 (2.8)	15.9 (2.5)	15.3 (3.1)	< .001	0.20

Notes: p-values for age and RSES total score are based on two-sample t-tests on the equality of mean; Cohen’s d is reported for effect size; p-values for literacy, numbers of children and household size are based on Mann-Whitney's U test on the equality of mean ranks, r is reported for effect size; Marital status difference is based on Cramer’s V Association.

Data was collected over an extended period from June to December 2017. The length of this period was in part due to the remoteness of fieldwork sites and the time-consuming process of selecting women for the control group through participatory wealth ranking exercises. Data collection was undertaken by the Social Impact team, which is a semi-autonomous monitoring and evaluation branch within *Fonkoze*.

Research adhered to ethical protocol, including informed consent, anonymity in data analysis and dissemination and respectful conduct in the field. All research respondents provided informed consent before participating in the study. They received verbal information (in Haitian Creole) about the research objectives and the requested input. Respondents were allowed to offer consent in the most culturally appropriate way, which in all cases proved to be verbal consent (due to high levels of illiteracy among research participants). Ethical clearance for this study was provided by the Research Ethics Committee of the Institute of Development Studies in March 2017.

### Measures

#### Rosenberg Self-Esteem Scale

The RSES is a 10-item measure of self-esteem [19], with a scoring range of 0–30, that includes half positively and half negatively worded statements such as “*On the whole*, *I am satisfied with myself*” and “*I certainly feel useless at times*”. It is the most widely used measure of self-esteem employed with adults and youth globally. It has been extensively validated with evidence for cultural variations in its constructs (the focus of this study). In this sample, internal consistency for the full 10-item scale was α = 0.52. This low alpha is consistent with several studies using the RSES as a single-factor structure in LMICs including Fromont, Haddad [24] in Burundi; Oladipo and Kalule-Sabiti [36] in Nigeria; and Schmitt and Allik [20] in the Democratic Republic of Congo, Ethiopia and Tanzania. It contrasts with better internal consistency found in a Costa Rica sample Li, Delvecchio [25] (see Table 1).

#### K6

The K6 is a 6-item self-report measure with a five-point response scale designed to screen for serious mental illness [21], and has been validated for use in multiple cultural contexts with very good specificity and sensitivity for psychological distress [37]. Items cover typical symptoms of psychological distress including feelings of hopelessness, nervousness, depression, and worthlessness. It has been adopted by the World Health Organisation for use in World Mental Health surveys and is therefore one of the most widely used screening tools for mental illness [38]. The measure has been translated into Haitian Creole and used in a Haitian population but not been formally validated in this context [39–41]. In this sample, the internal consistency was α = 0.83.

Table 3 provides a detailed overview of items included in the RSES and K6 measures.

**Table 3:** Items included in RSES and K6.

**Rosenberg Self-Esteem Scale (RSES)** *(strongly agree = 3*, *agree = 2*, *disagree = 1*, *strongly disagree = 0; items with an asterisk are reverse scored)*	**K6 scale** *(all = 1*, *most = 2*, *some = 3*, *a little = 4*, *none = 5)*
1. On the whole, I am satisfied with myself. 2. At times, I think I am no good at all.* 3. I feel that I have a number of good qualities. 4. I am able to do things as well as most other people. 5. I feel that I do not have much to be proud of.* 6. I certainly feel useless at times.* 7. I feel that I’m a person of worth, at least on an equal level with others. 8. I wish I could have more respect for myself.* 9. All in all, I am inclined to feel that I am a failure.* 10. I take a positive attitude toward myself.	1. About how often during the past 30 days did you feel nervous? 2. About how often during the past 30 days did you feel hopeless? 3. About how often during the past 30 days did you feel restless or fidgety? 4. About how often during the past 30 days did you feel so depressed that nothing could cheer you up? 5. About how often during the past 30 days did you feel that everything was an effort? 6. About how often during the past 30 days did you feel worthless?

### Statistical analysis plan

Following initial data cleaning and testing for normality, confirmatory factor analyses (CFA) were used to replicate previously published factor structures, and exploratory factor analysis (EFA), as needed, using Maximum Likelihood extraction method and Varimax rotation, to test for novel models. Items were forced into one or two factors, with and without item 8, as per previously published models.

CFA using MPlus 7.2 were then conducted to establish model fit with models suggested by EFA. Items with <40% (r^2^ < .4) of their variance accounted for by the overall construct were removed from the analysis. CFA typically involves the random splitting of a sample into two to allow testing and confirmation of a measurement model. As membership of the control and treatment groups had not been randomly allocated in the first instance and statistical differences had been found between the two groups on demographic and test variables, these groups were deemed unsuitable for use. Therefore, they were collapsed together and then randomly split. No differences were found between the two new groups on means for age, K6 or RSE total score, nor between medians for marital status, literacy, or number of children, making them suitable groups for CFA model testing and confirmation. Multiple fit indices were used to address limitations within individual fit indices: a comparative fit index (CFI) of >.90 is acceptable and >.95 is good; a Root Mean Square Error of Approximation (RMSEA) of < .06 is good, and < .05 (or a 95% confidence interval that was < .05) is very good; and a Standardized Root Mean Square Residual (SRMR) of < .08 is good (Hu & Bentler, 1999). The chi-square was also documented. Significant improvements in model fit were compared using the Satorra–Bentler scaled chi square statistic (S-B χ^2^) [42].

Correlational analyses and ANOVA were used to test the association between self-esteem (RSES) and serious mental illness (K6).

For CFA, estimating sample size is not straightforward. Whilst Everitt [43] suggest a ratio of 10xNitems, with small scales this can lead to under-estimation of required sample size. Comrey and Lee [44] suggest that 500 participants is a very good sample and that 1,000 or more is excellent. Based on Everitt’s guidance, and as the sample was split for the CFA, we doubled the Nx10 number, and in anticipation of conducting the analysis between the RSES and K6 as a path analysis, calculated Nitems = 16. The minimum required sample size was therefore 320. Our final sample size of 1,381, comfortably exceeded Comrey and Lee’s benchmark for excellence.

## Results

### Data preparation

There was no missing data. The sample had a mean RSES score of 15.6 (SD = 2.8). A slight positive skew was evident with significant results for the Kolmogorov-Smirnov test (.090, p < .001). The K6 had a mean score of 19.0 (SD = 4.1) and showed a similar trend with a significant Kolmogorov-Smirnov test (.060, p < .001). Tests of normality tend towards being over-conservative for large samples, and consequences for analysis tend to disappear in samples over N = 200 [45]. A slight positive skew was evident on visual inspection of the histogram (see S1 Fig), but both measures provided an otherwise normal distribution. There was no correlation between age and either of the measures.

As no published validation for the K6 in a Haitian sample could be found, we conducted a brief CFA on the scale. The six items loaded onto a single factor achieving a good or acceptable fit on three indices (CFI = 0.931, RMSEA = 0.074 (95%CI = 0.000–0.168), SRMR = 0.080), and a non-significant χ^2^ of 12.19 (df = 9, p = .203).

### Testing the RSES factor structure

**Fig 1:**
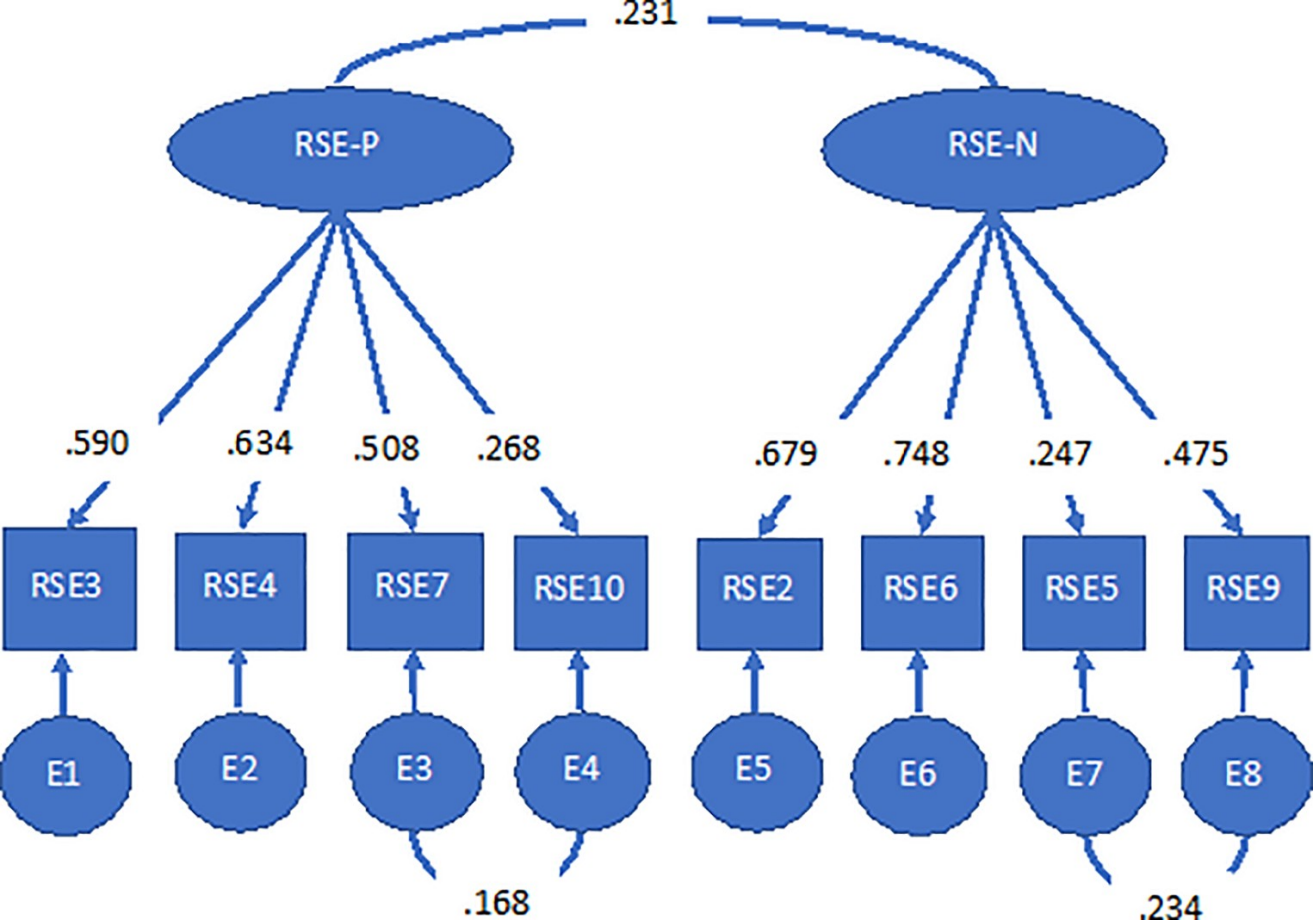
Final CFA model of RSE-8.

The factor analysis was conducted in two stages. Initially, we attempted to replicate pre-existing CFA of the RSES. The data did not fit any of these models (see Table 4). In the second stage, data was subjected to exploratory factor analysis to establish grounds for a novel model which could be tested through CFA. Items 1 and 8 failed to load on any factors, and were removed before repeating EFA. This time two factors emerged with four items loading onto each factor (see Table 5), both with eigen values >1 accounting cumulatively for 48.6% of the variance. There was clear delineation between positively and negatively worded items. This was then tested with CFA using a split-half sample. This model achieved good overall fit (CFI = 0.940, RMSEA = 0.061 (CI = 0.044–0.078), SRMR = 0.040) with co-variances allowed between two items within each factor and between the two factors (see Fig 1). The model remained within acceptable parameters for all fit indices when re-tested in the other half of the sample, confirming this was the best fitting model.

**Table 4:** Fit-indices for competing RSES models.

Model	S-B χ2 (df)	CFI	RMSEA	SRMR
**1. 10-item 1-factor**	406.47 (35)	0.517	0.125	0.097
**2. 10-item 2-factor**	376.92 (34)	0.554	0.122	0.096
**3. 9-item 1-factor**	379.33 (27)	0.530	0.139	0.103
**4. 9-item 2-factor**	350.73 (26)	0.567	0.136	0.103
**5. 8-item 2-factor**	59.32 (17)	0.940	0.061	0.040

**Table 5:** Factor loadings for unconstrained EFA.

*Item*	*Factor 1*	*Factor 2*
2	.69	
6	.69	
9	.54	
5	.35	
7		.59
4		.59
3		.59
10		.36

Internal consistency for this new 8-item scale with two sub-scales was tested with Cronbach’s alpha. These were: RSE-8 Positive = .61, RSE-8 Negative = .65 (both questionable) and RSE-8 Total = .58 (poor). The sub-scales are therefore recommended for use in preference to the total scale.

### Discriminant validity

The new sub-scales scores were compared with the K6 total score to assess the extent to which the RSE-8 represented a distinct construct from psychological wellbeing. The RSE-8 Positive was significantly correlated with the K6, but the size of the correlation was negligible (r = .08, p = .005), with statistical significance reflecting the very large sample size rather than a meaningful relationship between the two variables. By contrast the RSE-8 Negative was significantly correlated with a medium effect size (r = .32, p < .001). This suggests that there is an association between negative self-esteem and serious mental illness but the size of the effect suggests that they are distinct constructs. The two sub-scales were not significantly associated with each other (r = -.06, ns), suggesting they measure unique constructs.

### Self-esteem as a predictor of mental health

**Fig 2:**
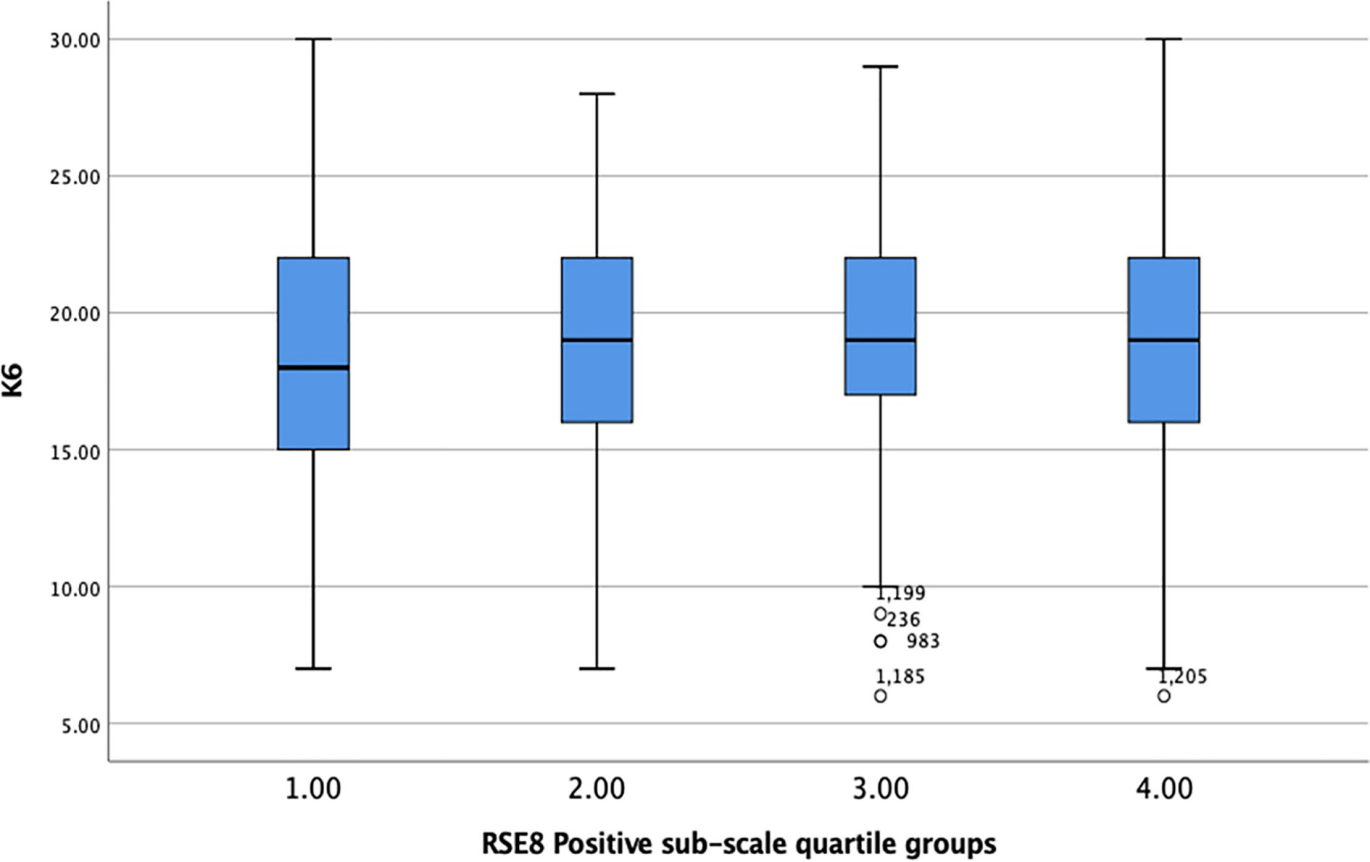
Box plot showing K6 scores plotted against quartile groupings of the RSE-8 Positive sub-scale.

**Fig 3:**
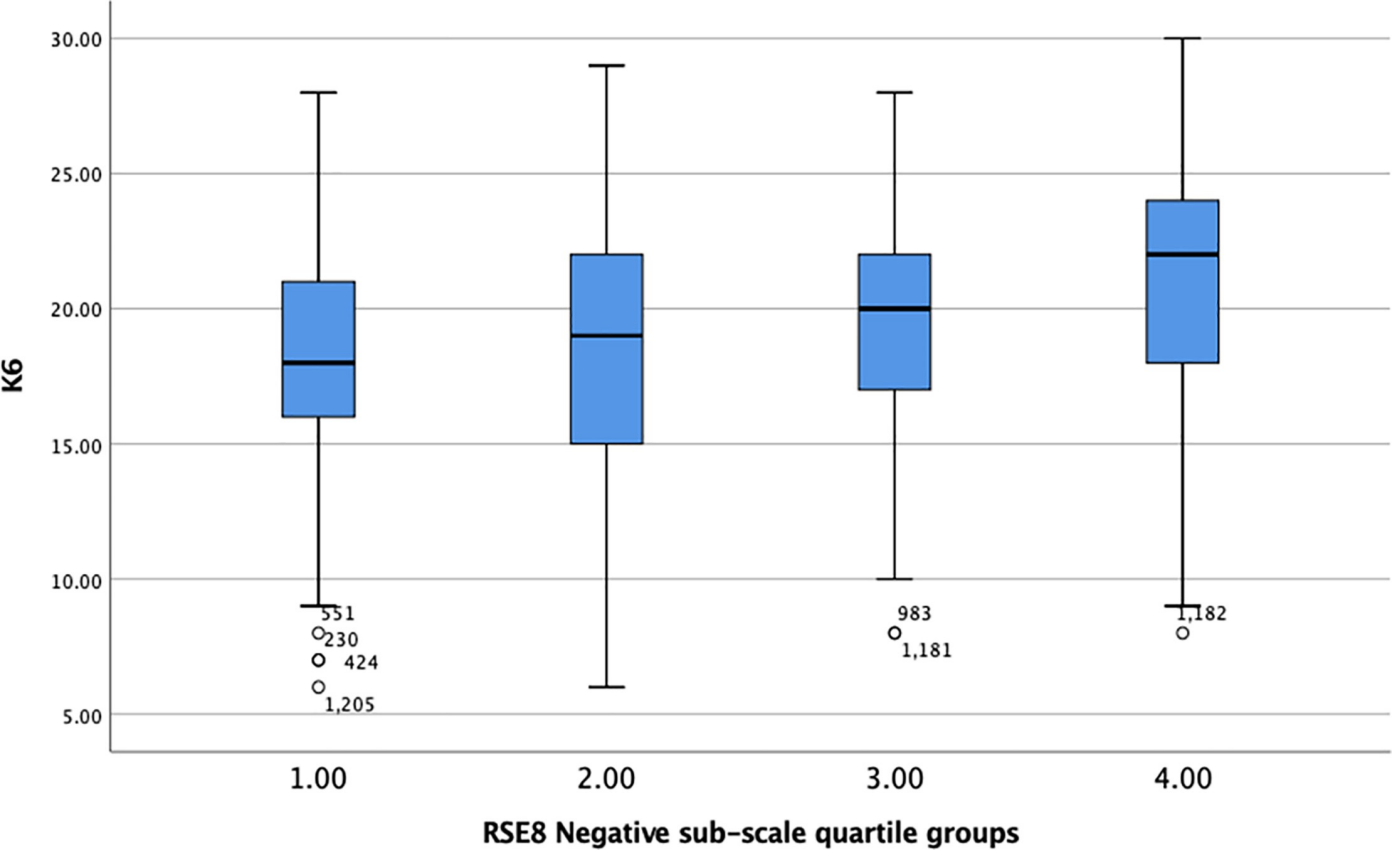
Box plot showing K6 scores plotted against quartile groupings of the RSE-8 Negative sub-scale.

Quartile groups were created for each RSE-8 sub-scale, and K6 scores plotted against these groups (see Figs 2 and 3).

One-way ANOVA found a significant difference between groups for the K6 and the RSE-8 Positive sub-scale (F = 3.31, df = 1380, p = 0.019, η^2^ = .007) but the effect size was marginal, explaining less than 1% of the variance between the groups. This is reflected in the box plots which shows an almost flat pattern with a slight curve suggesting that those in the mid-range for positive self-esteem had the highest levels of mental health problems compared to those with low or high self-esteem.

The RSE-8 Negative subscale showed a clearer pattern in relation to the K6 with negative self-esteem increasing in line with mental health difficulty. A significant difference was found between groups (F = 35.68, df = 1380, p < .0001, η^2^ = .072) with self-esteem predicting 7% of the difference in K6 scores. This represents a small but noticeable effect.

These findings suggest that whilst the negative sub-scale is clearly associated with mental health, this is a small association. The positive self-esteem sub-scale shows no such effect. Overall, the hypothesis that self-esteem, as measured by the RSES, can be used as a proxy measure for mental health is not supported.

## Discussion

In this study we set out to test the validity of the RSES and its applicability as a proxy measure for mental health within a sample of women living in rural Haiti. We did so against the backdrop of expanding interest in studying the negative cycle between poverty and mental health problems and potential solutions, and the concurrent need for robust and validated measures of mental health for doing so.

We find no evidence for a one-dimensional 10-item factor structure of RSES for adult women in rural Haiti. Instead, we find a two-dimensional 8-item factor structure with sub-scales for positively and negatively worded items that meets multiple fit indices in CFA, although the internal consistency of the overall structure and sub-scales is weak. Comparisons with the K6 measure of psychological distress display no significant correlation with the RSE-8 Positive sub-scale. They do show significant association with the RSE-8 Negative sub-scale, thereby endorsing the scale as a measure of low self-esteem. However, effect size suggests that the RSE-8 negative sub-scale and K6 are distinct constructs. Similarly, lack of association between RSE-8 positive and negative sub-scales means that they do not reflect the same constructs. In short, the applicability of the RSES as a measure of self-esteem for adult women in Haiti may be limited, and it should not be used as a proxy for mental health. It has psychometric properties that allow use in complex statistical analysis where its internal structure can be mapped as part of the analysis, e.g. path analyses.

Follow-up conversations with *Fonkoze* programme staff and enumerators allowed for further reflection on cross-cultural applicability of individual items. In line with research in other LMICs [20,24,26,36], item 8: *“I wish I could have more respect for myself”* did not work well in the Haitian context. Although a new round of two-way translation from English to Haitian Creole and back to English revealed that the question had been accurately translated, its interpretation was likely to be different from its intended meaning. Programme staff and enumerators indicated that the statement could have been interpreted in a rhetorical manner, with respondents indicating: “Well of course I would like to respect myself more”. Indeed, the large majority of respondents (94.7%) indicated to agree or strongly agree with this statement. Item 1: *“On the whole*, *I am satisfied with myself”* also did not load on any factors in our sample. The reason for this is less clear. Two-way translation confirms that the meaning and interpretation of this statement is largely the same in Haitian Creole. Descriptive analysis and further conversations with programme staff suggest that the item may be too ambiguously worded.

The apparent cultural differences in applicability of the RSES may reflect that self-esteem is a culture-specific construct. Other research in the Haitian context has also questioned the universal applicability of the self-esteem construct and the RSES as a measure for capturing this construct. A study with urban Haitian women at risk of HIV-infection found that feelings of self-worth and a sense of self were strongly related to other’s perceptions of them and the ability to care for their family [46]. In contrast to our findings, discussions with clinic staff and attendees during formative stages of the research suggested that negative and self-deprecating items in the RSES such as *“I feel I do not have much to be proud of”* may not be culturally appropriate (ibid). As a result, the RSES was not administered in this study. Others have highlighted that concepts of personhood in Haiti are multifaceted and that Haitians’ conceptualisation and communication of mental health is highly localised and culture-specific [47]. At present, few assessments in Haiti use culturally appropriate language, hampering efforts to expand understanding of mental health outcomes and guide models for mental health care [48].

## Conclusions

Our findings lend weight to the hypothesis that self-esteem is a culture-specific construct that cannot be assumed to be universal. It adds to a small but substantial body of literature from Haiti that highlights the need for more localised and contextualised understandings of self-esteem and mental health and illness more broadly. Further research is needed to localise self-esteem in the lives of women living in extreme poverty in rural Haiti, and to develop and validate a culturally relevant measure of self-esteem.

More broadly, our results indicate that caution is warranted when using RSES in different cultural contexts and with samples with different socioeconomic characteristics. Given the limits to cross-cultural applicability of the RSES and its use as a proxy measure, scrutiny is needed when using psychometric scales that were designed and validated in (mostly) high-income countries with very different populations.

This is particularly pertinent within the burgeoning field of studies assessing linkages between poverty and mental health and widening interest in impacts of anti-poverty interventions on mental health and psychological wellbeing. With studies including many measures and indicators, they tend to rely on psychometric validation undertaken in unrelated contexts and populations. As a result, studies risk under- or over-reporting results, drawing inaccurate conclusions or de-contextualising findings. Greater consideration of the validity of psychosocial constructs and their measurement is crucial for studies to offer robust and replicable insights into how the cycle between poverty and mental health problems may be broken.

## Supporting information

S1 Fig Fig 1 –Test of normality RSES. (TIF)

S1 Dataset Fonkoze CLM programme*–*Baseline data 2017. (DTA)
