# Supplementary figures and images for: Assessing mental health in a context of extreme poverty: Validation of the rosenberg self-esteem scale in rural Haiti
Source: PLoS One. 2020 Dec 14;15(12):e0243457. doi: 10.1371/journal.pone.0243457 (PMC7735634; doi:10.1371/journal.pone.0243457)

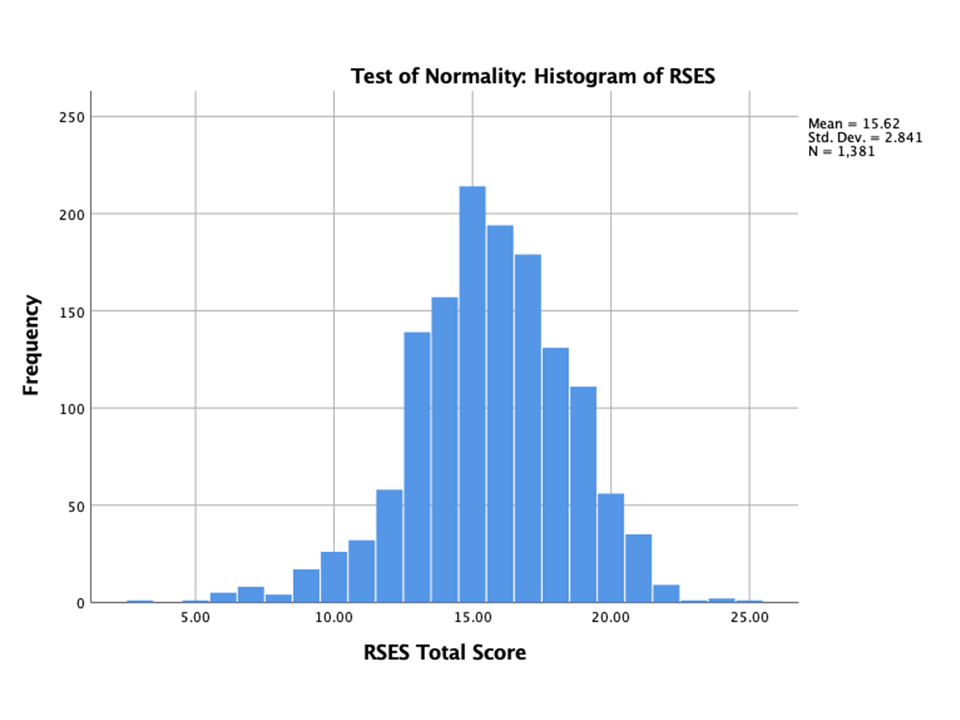

Supplement: S1 Fig — (TIF) [file pone.0243457.s001.tif]
